# Associations of 24 h time-use compositions of sitting, standing, physical activity and sleeping with optimal cardiometabolic risk and glycaemic control: The Maastricht Study

**DOI:** 10.1007/s00125-024-06145-0

**Published:** 2024-04-24

**Authors:** Christian J. Brakenridge, Annemarie Koster, Bastiaan E. de Galan, Alison Carver, Dorothea Dumuid, Francis Q. S. Dzakpasu, Simone J. P. M. Eussen, Hans H. C. M. Savelberg, Hans Bosma, Neville Owen, Nicolaas C. Schaper, Genevieve N. Healy, David W. Dunstan

**Affiliations:** 1 Baker Heart and Diabetes Institute, Melbourne, VIC Australia; 2 Mary Mackillop Institute for Health Research, Australian Catholic University, Melbourne, VIC Australia; 3 Active Life Lab, South-Eastern Finland University of Applied Sciences, Mikkeli, Finland; 4 Centre for Urban Transitions, Swinburne University of Technology, Melbourne, VIC Australia; 5 Department of Social Medicine, Maastricht University, Maastricht, the Netherlands; 6 CAPHRI Care and Public Health Research Institute, Maastricht University, Maastricht, the Netherlands; 7 Department of Internal Medicine, Maastricht University Medical Center+, Maastricht, the Netherlands; 8 Department of Internal Medicine, Radboud University Medical Centre, Nijmegen, the Netherlands; 9 CARIM School for Cardiovascular Diseases, Maastricht University, Maastricht, the Netherlands; 10 National Centre for Healthy Ageing, The School of Translational Medicine, Monash University, Melbourne, VIC Australia; 11 Alliance for Research in Exercise, Nutrition and Activity, University of South Australia, Adelaide, SA Australia; 12 Department of Epidemiology, Maastricht University, Maastricht, the Netherlands; 13 Department of Nutrition and Movement Science, Maastricht University, Maastricht, the Netherlands; 14 NUTRIM School for Nutrition and Translational Research in Metabolism, Maastricht University, Maastricht, the Netherlands; 15 School of Human Movement and Nutrition Sciences, The University of Queensland, Brisbane, QLD Australia; 16 Institute for Physical Activity and Nutrition, School of Exercise and Nutrition Sciences, Deakin University, Melbourne, VIC Australia

**Keywords:** Cardiometabolic risk, Glycaemic control, Physical activity, Sitting, Sleep, Time use

## Abstract

**Aims/hypothesis:**

The associations of sitting, standing, physical activity and sleep with cardiometabolic health and glycaemic control markers are interrelated. We aimed to identify 24 h time-use compositions associated with optimal metabolic and glycaemic control and determine whether these varied by diabetes status.

**Methods:**

Thigh-worn activPAL data from 2388 participants aged 40–75 years (48.7% female; mean age 60.1 [SD = 8.1] years; *n*=684 with type 2 diabetes) in The Maastricht Study were examined. Compositional isometric log ratios were generated from mean 24 h time use (sitting, standing, light-intensity physical activity [LPA], moderate-to-vigorous physical activity [MVPA] and sleeping) and regressed with outcomes of waist circumference, fasting plasma glucose (FPG), 2 h plasma glucose, HbA_1c_, the Matsuda index expressed as *z* scores, and with a clustered cardiometabolic risk score. Overall analyses were adjusted for demographics, smoking, dietary intake and diabetes status, and interaction by diabetes status was examined separately. The estimated difference when substituting 30 min of one behaviour with another was determined with isotemporal substitution. To identify optimal time use, all combinations of 24 h compositions possible within the study footprint (1st–99th percentile of each behaviour) were investigated to determine those cross-sectionally associated with the most-optimal outcome (top 5%) for each outcome measure.

**Results:**

Compositions lower in sitting time and with greater standing time, physical activity and sleeping had the most beneficial associations with outcomes. Associations were stronger in participants with type 2 diabetes (*p*<0.05 for interactions), with larger estimated benefits for waist circumference, FPG and HbA_1c_ when sitting was replaced by LPA or MVPA in those with type 2 diabetes vs the overall sample. The mean (range) optimal compositions of 24 h time use, considering all outcomes, were 6 h (range 5 h 40 min–7 h 10 min) for sitting, 5 h 10 min (4 h 10 min–6 h 10 min) for standing, 2 h 10 min (2 h–2 h 20 min) for LPA, 2 h 10 min (1 h 40 min–2 h 20 min) for MVPA and 8 h 20 min (7 h 30 min–9 h) for sleeping.

**Conclusions/interpretation:**

Shorter sitting time and more time spent standing, undergoing physical activity and sleeping are associated with preferable cardiometabolic health. The substitutions of behavioural time use were significantly stronger in their associations with glycaemic control in those with type 2 diabetes compared with those with normoglycaemic metabolism, especially when sitting time was balanced with greater physical activity.

**Graphical Abstract:**

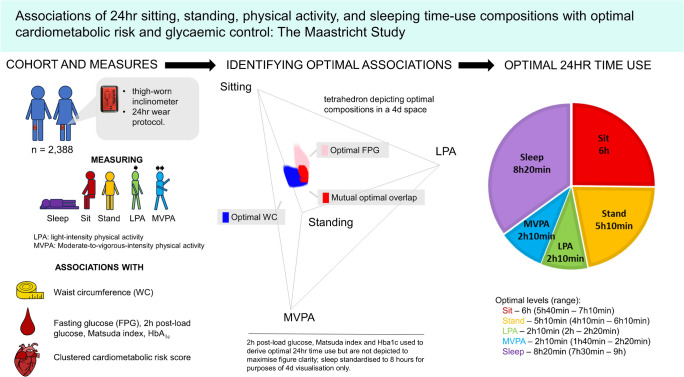

**Supplementary Information:**

The online version contains peer-reviewed but unedited supplementary material available at 10.1007/s00125-024-06145-0.


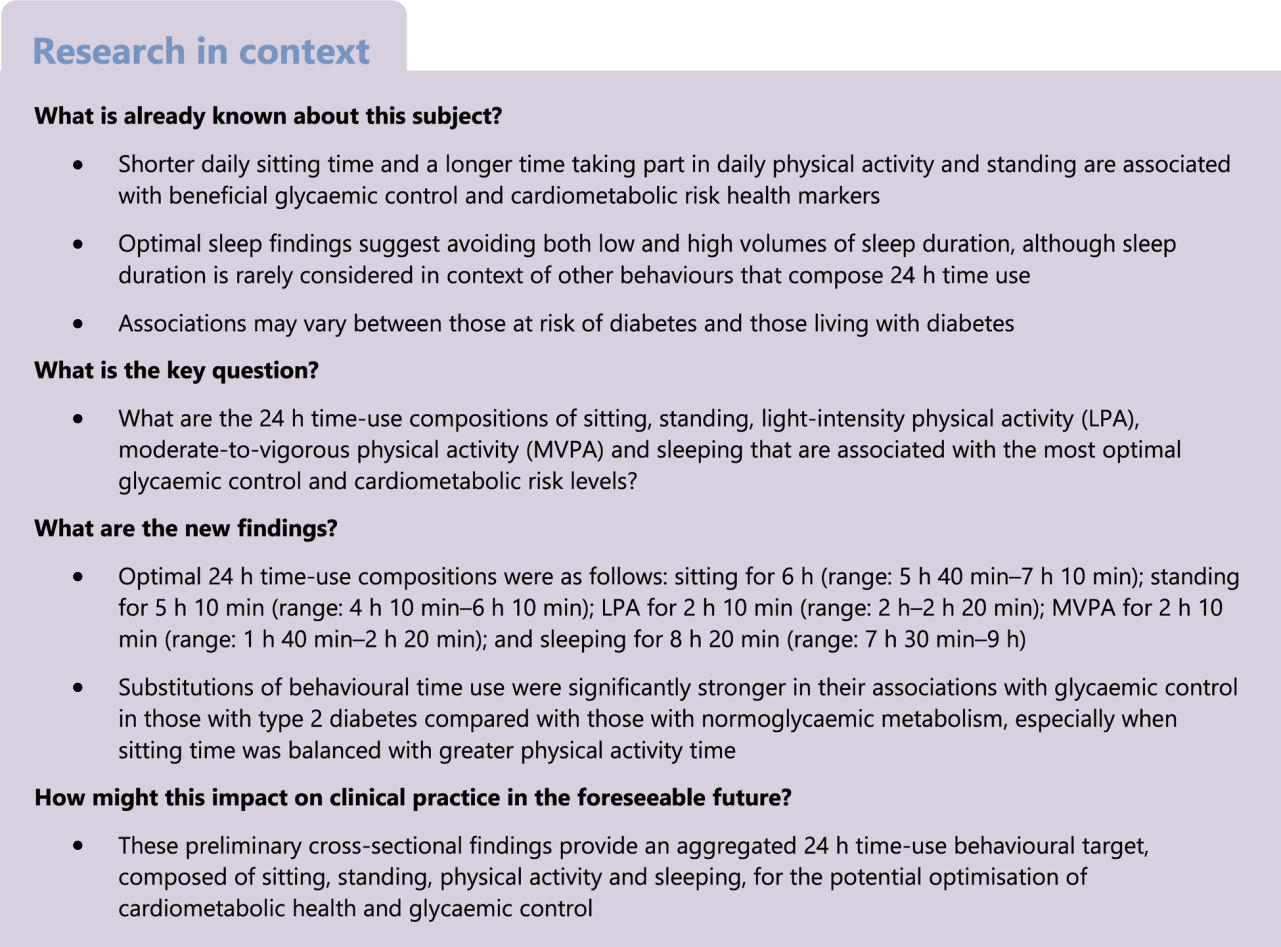


## Introduction

Guidelines for 24 h movement [1, 2] are based on evidence that behaviours composing a day (sitting, standing, physical activity and sleep) can have interrelated contributions to health. Changing time spent in one of these behaviours will necessarily change the time spent in another. While the 24 h guidelines have been informed by a broad body of evidence [3], a commonly referenced limitation is a lack of relevant findings from studies employing a compositional analytic approach [2, 4]. Identifying the optimal balance of 24 h behavioural time-use compositions (sitting, standing, physical activity and sleeping) and the relationships of indicators of cardiometabolic health and glycaemic control with compositional techniques, can further inform 24 h guidelines and provide more precise targets for the improvement of disease risk and management of diseases such as type 2 diabetes.

Continuous measurement approaches, such as those collected via thigh-worn accelerometers, facilitate the investigation of 24 h free-living behaviours. Compositional data analysis (CoDA) appropriately considers the time spent in these behaviours as relative to one another and as having interrelated influence on health outcomes. Although well established in other fields, such as geochemistry [5] and nutrition [6], the application of CoDA is relatively new to physical activity and sedentary behaviour fields [7]. There has been limited application of this methodology in understanding different risk profiles, including in people with, or at risk of, type 2 diabetes [4, 8]. There is a need to evaluate the health risks of excess sedentary behaviour [9], low physical activity [10, 11] or inadequate sleep [12] as having interrelated implications for disease risk and disease management.

To address the evidence gaps, we examined associations of compositions of sitting, standing, light-intensity physical activity (LPA), moderate-to-vigorous physical activity (MVPA) and sleep time with cardiometabolic risk and glycaemic control markers in a large sample of adults using thigh-worn accelerometers. Associations were examined overall, as well as by diabetes status (normoglycaemia, impaired glucose metabolism [IGM], type 2 diabetes) and sex. The compositions associated with more-optimal benefits, for all cardiometabolic and glycaemic control markers, were also investigated. It was hypothesised that compositions with longer sitting time would be adversely associated with cardiometabolic parameters, while longer standing and physical activity time would be beneficially associated with cardiometabolic parameters, and that these associations would be stronger in people with type 2 diabetes and IGM than in those with normoglycaemia.

## Methods

### Study population

Data were obtained from The Maastricht Study [13], which is an ongoing observational study of adults aged between 40 and 75 years old living in the Southern Netherlands. The rationale and study methodology have been described previously [13]. In brief, recruitment was conducted through mass media campaigns, municipal registries and the regional diabetes patient registry. Participants were recruited and stratified according to diabetes status to investigate the aetiology and pathophysiology of diabetes, with an oversampling of those with known type 2 diabetes status. The current report includes cross-sectional data from 3451 participants recruited between November 2010 and September 2013; all examinations were performed on each participant within 3 months of consent. Exclusions were applied if they had missing data on the following covariates: sex; age; education category; smoking status; and adherence to the Dutch Healthy Diet index (*n*=334) [14]. Participants who had invalid activPAL activity monitor data (*n*=100) or did not wear the device (*n*=601) were excluded. Participants with type 1 diabetes or latent autoimmune or steroid-induced diabetes, or diabetes following pancreatectomy, were also excluded (*n*=28), leaving 2388 participants for the present analyses. Ethnicity data were collected; based on self-report, nearly all participants were of European descent [13].

The Maastricht Study was approved by the institutional medical ethical committee (NL31329.068.10) and the Minister of Health Welfare and Sports of the Netherlands (permit no. 131088-105234-PG). Written informed consent was obtained from all participants. The manuscript was written in accordance with the Strengthening the Reporting of Observational Studies in Epidemiology (STROBE) reporting guidelines [15].

### Sitting, standing, physical activity and sleeping

Daily behaviours (sitting, standing, stepping and sleeping) were measured using the activPAL3 inclinometer (version 6.4.1; PAL Technologies, Glasgow, UK). The device was attached directly to the skin on the front of the right thigh with transparent tape and was waterproofed with a nitrile sleeve. Participants were instructed to wear the monitor continuously for eight consecutive days without removal. To avoid inaccurately identifying non-wear time, participants were asked not to replace the device once removed. Data were uploaded using the activPAL software and processed using customised software written in MATLAB R2018b (MathWorks, Natick, MA, USA). The first measured wear day was excluded from analyses because it coincided with the clinical assessment and was therefore not a true representation of typical-day behaviours. Data from the final wear day, containing ≤14 h information, were also excluded for each participant. Data were included for analyses if there was at least one valid wear day that constituted ≥14 h of waking time. Stepping minutes were further categorised into LPA (<100 steps/min) and MVPA (≥100 steps/min) according to common cadence definitions [16]. The total amount of time spent sitting, standing, and in LPA and MVPA was divided by the number of valid wear days to derive average daily totals. Average daily sleeping time was estimated by subtracting the average waking time use from 24 h. An automated algorithm was used to determine sleep and waking time, as described elsewhere [17], therefore misclassification was possible (e.g. time lying in bed in some instances may have been incorrectly classified as sleeping time).

### Cardiometabolic risk-marker outcomes

Outcome measures were waist circumference, fasting plasma glucose (FPG), 2 h post-load glucose (2hPLG), HbA_1c_, Matsuda index (ISI-M) and a clustered cardiometabolic risk score (CMR). The normality of residuals for FPG, 2hPLG and HbA_1c_ improved following natural logarithm transformation. Waist circumference was measured manually with a tape measure midway between the lower rib margin and the peak of the iliac crest to the nearest 0.5 cm. Fasting samples were assessed using a standard enzymatic hexokinase reference method for plasma glucose. HbA_1c_ was measured with ion-exchange HPLC. All included participants underwent a standardised 2 h OGTT, as described previously [13], where blood draws subsequent to fasting were collected at 15, 30, 45, 60, 90 and 120 min. The 2hPLG was informed by the OGTT and all six time points of glucose and insulin concentrations informed the calculation of the ISI-M, with a higher index indicating higher insulin sensitivity [18]. The ISI-M was calculated using fasting and mean glucose and insulin values, as follows (where FPI is fasting plasma insulin):

$$\mathrm{ISI}-\mathrm M=\frac{10,000}{\sqrt{\left(\mathrm{FPG}\;\times\;\mathrm{FPI}\right)\;\times\;\left(\mathrm{mean}\;\mathrm{OGTT}\;\mathrm{glucose}\;\mathrm{concentration}\;\times\;\mathrm{mean}\;\mathrm{OGTT}\;\mathrm{insulin}\;\mathrm{concentration}\right)}}$$

The clustered CMR was calculated using five cardiometabolic markers, including waist circumference, FPG, triacylglycerol, HDL-cholesterol and average BP, as per previously devised methods [19, 20]. Fasting blood samples were used for HDL-cholesterol and triacylglycerol analyses, which were assessed in laboratory with enzymatic and/or colorimetric methods by an automatic analyser (Beckman Synchron LX20; Beckman Coulter, Brea, CA, USA). Average systolic and diastolic BP was calculated from three office measurements of the right arm after a 10 min rest period using a non-invasive BP monitor (OMROW 705IT; OMRON, Kyoto, Japan). The average BP outcome was calculated by adding the systolic and diastolic measures together and dividing the value by two. Triacylglycerol, HDL-cholesterol and FPG measures were log-transformed. All variables were then standardised according to the mean [*z*=(value–mean)/SD]. The risk score was then calculated by summing all the scores (with HDL-cholesterol added in inverse) and dividing the sum by five. Higher CMR is relative to the sample mean and is indicative of higher cardiometabolic disease risk [19].

### Covariates

Covariates were extracted from questionnaires administered during baseline assessment and included sex (self-reported), age, education (low, medium, high), smoking history (never, former, current smoker) and diet quality score. Education was ascribed as follows: low if the participant’s highest education was no education, primary education or lower vocational education; medium if it was general secondary education, general vocational education or higher secondary and pre-university education; and high if it was higher vocational education or university. Diet quality was measured with a validated Food Frequency Questionnaire [21] and scored as 0–140 using a Dutch Health Diet Index as a measure of adherence to the Dutch dietary guidelines, with higher scores indicating greater adherence [22]. Linear regression models featured adjustment for waist circumference, except where the independent variable was waist circumference or CMR. Diabetes status was assessed according to WHO 2006 criteria [23] using results from a 2 h OGTT and if the participant were using glucose-lowering medication. IGM was defined as impaired glucose tolerance (FPG <7.0 mmol/l and 2hPLG between ≥7.8 and <11.1 mmol/l) and/or impaired fasting glucose (FPG between 6.1 and 6.9 mmol/l and 2hPLG <7.8 mmol/l). Type 2 diabetes was defined as FPG ≥7.0 mmol/l and 2hPLG ≥11.1 mmol/l. Normal glucose metabolism was defined as below the IGM and type 2 diabetes cut points.

### Statistical analyses

All analyses were conducted using R statistical analysis software version 4.0 (R Foundation for Statistical Computing, Vienna, Austria). In CoDA [7], the outcome is dependent on compositional isometric log ratios (ilrs) multiplied by their coefficients and then summed with covariates in a linear regression model. Within this model, ilrs map compositional data into real space. There were no behaviour counts equalling zero for sitting, standing, physical activity or sleeping. Behaviours were first transformed into a finite composition using the ‘acomp’ function in R package Compositions [24]. To investigate the associations between time use (expressed as ilrs) and the chosen outcome variables, linear regression was performed. A five-part composition can be expressed as a set of four ilrs (i.e. ilr1, ilr2, ilr3, ilr4), which were included in all analyses. To test the association of increasing sitting relative to remaining behaviours, the first ilr (corresponding to the β_1_ coefficient in the regression model) was constructed to reflect the effect of time sitting relative to the other three behaviours. Therefore, ilr1 is equal to:

$$\mathrm{ilr}1\;\left(\mathrm{sitting}\;\mathrm{vs}\;\mathrm{standing},\;\mathrm{LPA},\;\mathrm{MVPA},\;\mathrm{sleeping}\right)\;=\;\sqrt{\left(\frac45\right)}\log_{\mathrm e}\left(\frac{\mathrm{sitting}}{4\sqrt{\left[\mathrm{standing}\;\times\;\mathrm{LPA}\;\times\;\mathrm{MVPA}\;\times\;\mathrm{sleeping}\right]}}\right)$$

The behaviours in the ilrs can be reordered in regression modelling as per the permutation principle [7] and give the same fit regardless of order. This allows each behaviour to be explored relative to the remaining behaviours. The composition (the set of ilrs in the regression model) was investigated for interaction by sex and diabetes status and the moderation on ilr1 coefficient was reported. The ilr models were used to perform isotemporal substitution (with R package: deltacomp [25]) whereby 30 min in one behaviour were substituted for 30 min in another. This method, explained in detail elsewhere [26], produces an estimated difference in the risk-marker value when time is reallocated to/from the geometric mean.

The optimal composition associated with each outcome was predicted by feeding a range of simulated compositions rounded to the nearest 10 min into the regression models. The simulated compositions were restricted to be within the empirical footprint, varying from the first to the 99th percentile of each behaviour. The full range of simulated compositions was created by producing every combination of these behaviours with one another that summed to 24 h. This resulted in 142,938 possible composition permutations for the overall sample. The compositions associated with the best 5% of *z* scores (i.e. lowest cardiometabolic risk) were chosen as the range of optimal compositions. The area at which the optimal compositions (by health outcome) overlapped in compositional space was taken as the unanimous overlapping optimal composition of 24 h time use. In many instances the overlapping compositions required that the optimal compositions be extended beyond the top 5% (i.e. to top 10%) to have a mutually overlapped area between outcomes in compositional space. Tetrahedrons were used to visualise the behavioural dynamics and overlapping optimal space, with sleep fixed at 8 h and the remaining behaviours preserved in four dimensions to represent waking time only. These were produced using the R package: rgl [27].

## Results

Table 1 shows the characteristics of the 2388 participants, overall and stratified by diabetes status. Overall, the sample was evenly balanced between male (51.3%) and female sex (48.7%). In addition, a former smoking history was most common (52%), as was high education level (38.3%) in the sample. Sitting time occupied the greatest proportion of the day, and sleeping time was similar across all strata. The IGM and type 2 diabetes groups had higher sitting levels, and lower standing and physical activity levels compared with the normoglycaemic group.

**Table 1:** Characteristics of participants stratified by diabetes status

Characteristic	Overall population	NGM	IGM	Type 2 diabetes
Participants, *n*	2388	1341	363	684
Age, years	60.1±8.1	58.2±8.1	62.1±7.2	62.7±7.7
Sex, *n* (%)				
Male	1224 (51.3)	548 (40.9)	196 (54.0)	480 (70.2)
Female	1164 (48.7)	793 (59.1)	167 (46.0)	204 (29.8)
Education, *n* (%)				
Low	802 (33.6)	359 (26.8)	133 (36.6)	310 (45.3)
Medium	671 (28.1)	381 (28.4)	96 (26.4)	194 (28.4)
High	915 (38.3)	601 (44.8)	134 (36.9)	180 (26.3)
Smoking history, *n* (%)				
Never	847 (35.5)	539 (40.2)	105 (28.9)	203 (29.7)
Former	1241 (52.0)	643 (47.9)	219 (60.3)	379 (55.4)
Current	300 (12.6)	159 (11.9)	39 (10.7)	102 (14.9)
Use of glucose-lowering medication, *n* (%)	545 (22.8)	0 (0.0)	0 (0.0)	545 (79.7)
Diet score	83.8±14.7	85.7±14.4	82.9±14.9	80.5±14.6
Waist circumference, mean cm (SD range)	94.8 (86.0, 104.0)	89.3 (82.3, 97.3)	97.8 (90.2, 105.0)	104.7 (96.5, 114.0)
FPG, mmol/l	5.5 (5.1–6.6)	5.1 (4.9–5.5)	6.0 (5.5–6.3)	7.6 (6.8–8.6)
2hPLG, mmol/l	6.30 (5.1–9.4)	5.4 (4.6–6.2)	8.4 (7.4–9.4)	14.5 (12.0–17.2)
HbA_1c_, mmol/l	38.0 (35.0–44.0)	36.0 (34.0–38.0)	38.0 (35.0–42.0)	50.0 (45.0–56.0)
HbA_1c_, %	5.6 (5.4–6.2)	5.4 (5.3–5.6)	5.6 (5.4–6.0)	6.7 (6.3–7.3)
ISI-M	3.44 (2.0–5.2)	4.4 (3.1–6.1)	2.6 (1.6–3.5)	1.9 (1.2–3.0)
CMR	−0.1 (−0.5–0.5)	−0.4 (−0.8–0.0)	0.1 (−0.2–0.5)	0.7 (0.3–1.0)
Valid activPAL days, *n*	6.3±1.2	6.3±1.1	6.3±1.1	6.1±1.3
Behaviour in 24 h, h				
Sitting	9.4 (8.3–10.6)	9.1 (8.0–10.1)	9.6 (8.3–10.6)	10.2 (9.1–11.2)
Standing	4.2 (3.4–5.1)	4.4 (3.7–5.3)	4.2 (3.3–5.0)	3.7 (3.0–4.7)
LPA	1.1 (0.8–1.3)	1.1 (0.9–1.4)	1.1 (0.8–1.3)	0.9 (0.7–1.2)
MVPA	0.9 (0.6–1.2)	1.0 (0.7–1.2)	0.8 (0.6–1.1)	0.6 (0.4–1.0)
Sleeping	8.2 (7.7–8.9)	8.2 (7.7–8.8)	8.2 (7.7–8.7)	8.3 (7.6–9.0)

Data are presented as mean±SD for normally distributed measures or median (IQR) for non-normally distributed measures, unless stated otherwise

### Compositional isotemporal substitution modelling

**Fig. 1:**
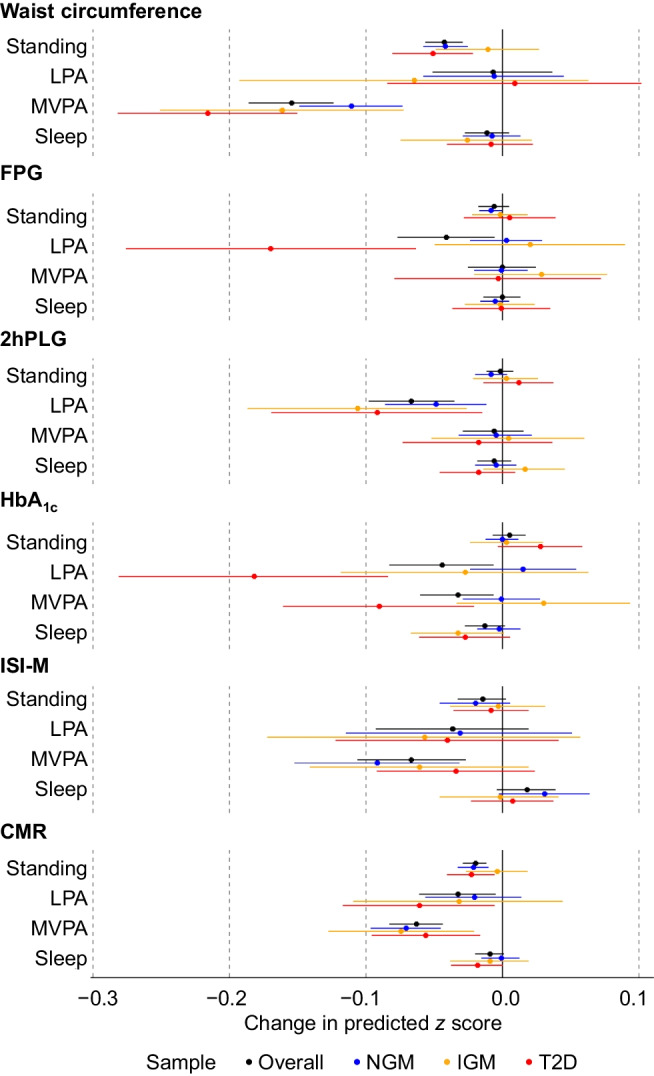
Estimating the difference incurred to glycaemic control and cardiometabolic risk markers with isotemporal substitution of 30 min from sitting to standing, physical activity and sleeping. Models are adjusted for age, sex, education, smoking status and dietary intake score. Overall sample analysis additionally adjusted for diabetes status. NGM, *n*=1341; IGM, *n*=363; type 2 diabetes, *n*=684. T2D, type 2 diabetes

Figure 1 depicts the estimated difference from the mean for risk markers when substituting 30 min of time spent sitting with other behaviours; all pairwise substitutions are depicted in electronic supplementary material (ESM) Tables 1–6. In the overall sample, higher LPA or MVPA levels with lower levels of sitting had beneficial associations with all risk markers, with variation as to whether the marker favoured higher compositional LPA (e.g. FPG) or MVPA (e.g. waist circumference). Higher standing time with lower levels of sitting time was significantly beneficially associated with waist circumference (*z* score [95% CI]: −0.04 [−0.06, −0.03]) and CMR (*z* score [95% CI]: −0.02 [−0.03, −0.01]) only. More time use spent sleeping was associated with benefit, for HbA_1c_ when replacing standing time (*z* score [95% CI]: −0.02 [−0.04, −0.00]), otherwise it had adverse associations when replacing LPA, MVPA or non-significant associations. The estimated difference in risk markers was most pronounced in the type 2 diabetes group, especially when compared with the normoglycaemic group, for FPG and HbA_1c_ and when LPA or MVPA replaced sitting. When MVPA replaced LPA this was associated with higher FPG in the type 2 diabetes group only. Basic compositional regression modelling (ESM Table 7) was additionally performed to investigate interactions by sex (ESM Table 8) and diabetes status (ESM Tables 9 and 10), and adjustment by waist circumference (ESM Table 11), on the relationships. Only CMR differed significantly by sex, suggesting that the beneficial association of standing at the expense of other behaviours was stronger in female participants. Adjustment by waist circumference resulted in the attenuation of all effect estimates, though they remained statistically significant for 2hPLG, HbA_1c_ and ISI-M outcomes.

### Optimal compositions of time use

**Fig. 2:**
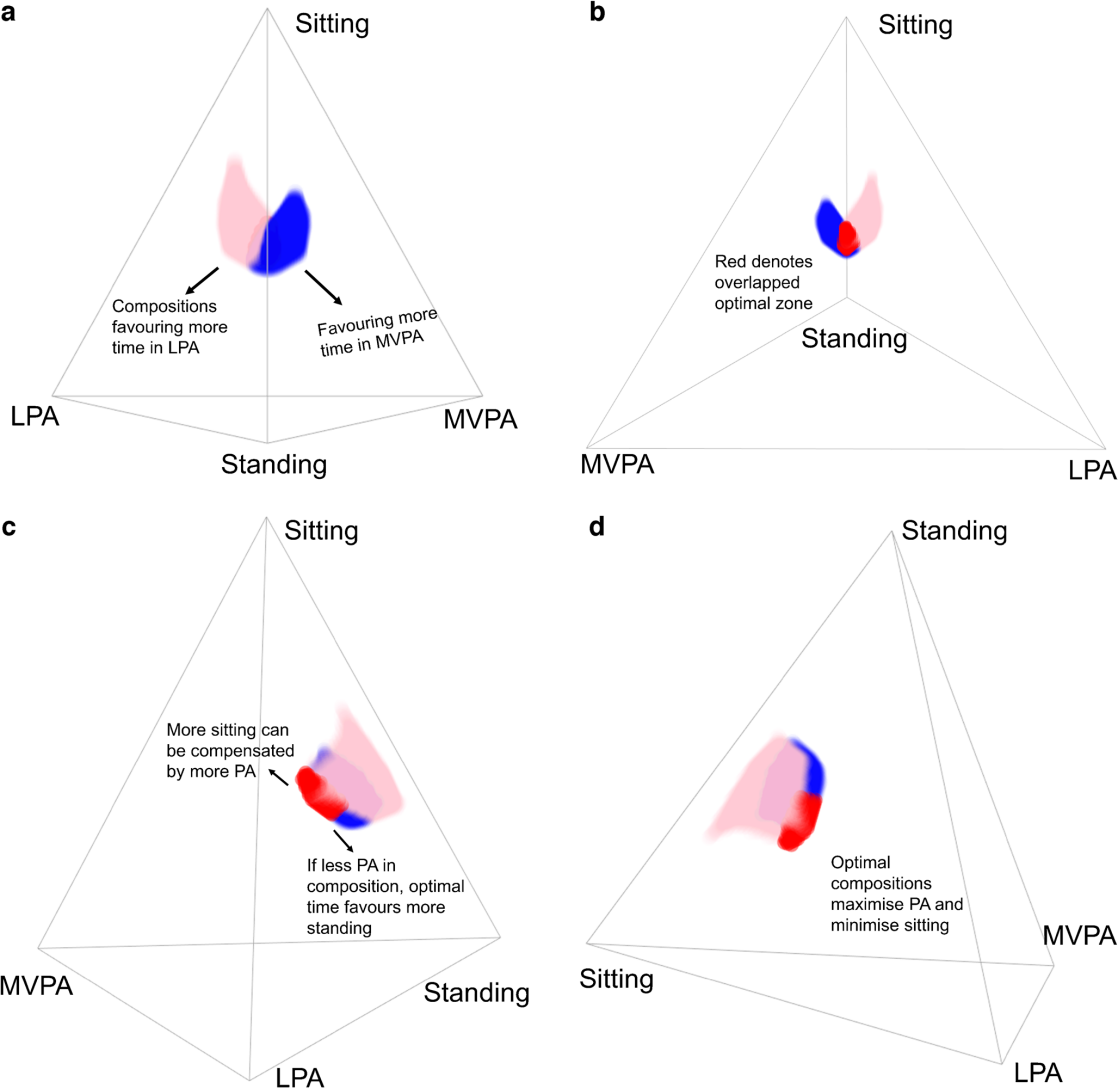
Optimal compositions of time use visualised in multidimensional space, adjusted for age, sex, education, smoking status, dietary intake score and diabetes status. Blue, waist circumference; pink, FPG; red, zone where all markers (including 2hPLG, HbA_1c_, ISI-M and CMR) share mutual overlap. Sleeping time was fixed to 8 h. Each corner of the tetrahedron depicts 100% of time use, and a composition data point directly in the middle of the tetrahedron would depict equal, 25% (4 h) of time use in sitting, standing, LPA and MVPA. Images in (**a**–**d**) depict the same four-axis quaternary tetrahedron rotated in different ways. Only FPG and waist circumference are displayed here for clarity, with the mutually shared overlap calculated using all health markers. PA, physical activity

The optimal compositions of time use associated with optimal glycaemic control and cardiometabolic risk markers are depicted in Table 2. Across the markers, the optimal sitting time was consistently lower than the sample mean, the optimal standing time was mostly higher, and the optimal LPA and MVPA time was also higher for most markers. Optimal sleeping time had more variation across health markers, with 2hPLG and HbA_1c_ having more beneficial associations with sleep duration. The optimal compositions of time use are visualised in multidimensional space in Fig. 2, with Fig. 2a–d featuring the same tetrahedron rotated at different perspectives. Figure 2a demonstrates the optimal compositions for FPG, favouring more time spent in LPA, and optimal compositions for waist circumference, favouring more time spent in MVPA. Figure 2b demonstrates the overlapping space between waist circumference and FPG forming the overlapped optimal zone. Figure 2c shows that the higher end of the optimal sitting range (i.e. 7 h and 10 min) must be accompanied by higher levels of physical activity, whereas the lower end of the sitting range comprises higher standing time and lower levels of physical activity. An interactive version of this plot can be viewed separately in ESM Fig. 1.

**Table 2:** Compositions of 24 h time use associated with the most-optimum levels of glycaemic control and cardiometabolic risk markers

Measure (*z* score)	Compositional centre of behaviour				
	Sitting	Standing	LPA	MVPA	Sleeping
Sample mean, h:min (1st–99th percentile range)^a^	9:20 (5:40–13:20)	4:10 (1:40–7:50)	1:00 (0:30–2:20)	0:50 (0:10–2:20)	8:10 (6:20–10:40)
Waist circumference	6:20 (5:40–8:50)	6:40 (4:30–7:50)	1:10 (0:30–2:20)	2:00 (1:20–2:20)	7:40 (6:20–10:40)
FPG	6:40 (5:40–10:20)	6:30 (4:30–7:50)	2:10 (1:40–2:20)	0:50 (0:10–2:20)	7:30 (6:20–10:40)
2hPLG	7:00 (5:40–10:50)	3:30 (1:40–7:50)	2:20 (1:50–2:20)	1:20 (0:10–2:20)	9:20 (6:20–10:40)
HbA_1c_	7:50 (5:40–11:30)	2:30 (1:40–5:20)	2:00 (1:00–2:20)	1:50 (0:30–2:20)	9:40 (6:20–10:40)
ISI-M	7:10 (5:40–10:20)	6:10 (2:40–7:50)	1:50 (0:30–2:20)	2:00 (0:50–2:20)	6:50 (6:20–8:30)
CMR	6:10 (5:40–7:50)	6:10 (3:00–7:50)	1:40 (0:30–2:20)	2:00 (1:00–2:20)	7:40 (6:20–10:40)
Overlapped optimal zone^b^	6:00 (5:40–7:10)	5:10 (4:10–6:10)	2:10 (2:00–2:20)	2:10 (1:40–2:20)	8:20 (7:30–9:00)

Data correspond to the compositional centre and range of the most-optimal (top 5%) compositions. Compositional centre was calculated within the most-optimal compositional area using the geometric mean of the five behaviour components (sitting, standing, LPA, MVPA and sleeping) for the overall sample

Data are presented as h:min (range), unless stated otherwise, with estimates rounded to the nearest 10 min. This may have resulted in combined estimates not forming exactly 24 h

All models are adjusted for age, sex, education, smoking status, dietary intake score and diabetes status

^a^Shows the geometric mean of the sample in h:min, as well as the range of compositions in the study footprint including time use from the first to the 99th percentile for each behaviour

^b^Compositional centre of the overlapped optimal zone required extending all compositions (from the optimum top 5%) to the optimum top 10%, to obtain data on mutual overlap between markers

There were small differences in optimal time-use composition by diabetes status (ESM Tables 12–14), which were potentially attributable to the innate differences between the optimal levels in each of the strata. For example, the top 5% of individuals with normoglycaemic metabolism (NGM) had substantially lower absolute waist circumference compared with those in the top 5% of people with type 2 diabetes. To summarise, the NGM group and the optimal compositions favoured much greater levels of standing and lower levels of sitting relative to the remaining groups. In the IGM group, standing time was less influential on the outcomes, as indicated by an expansive range. The type 2 diabetes group and optimal compositions on average favoured greater levels of sleeping time.

## Discussion

These are novel findings from compositional analyses of free-living sitting, standing, physical activity and sleeping time in a large cross-sectional sample of middle-aged and older adults recruited to oversample people with type 2 diabetes. We show that optimal compositions of time use involved substantially less time spent sitting, a greater time spent standing and a substantially greater time being physically active than the times being achieved on average for each of these activities by the participants in our study. Optimal sleeping time aligned with the sample mean. The optimal time-use zone did not substantially differ by diabetes status, although compositions with greater physical activity and less sitting were associated more strongly in both the IGM and type 2 diabetes group. This highlights the importance of considering all the behaviours that compose 24 h time use when managing cardiometabolic disorders. The mean time-use composition that universally covered the optimal association of all cardiometabolic risk and glycaemic markers was as follows: sitting, 6 h (range: 5 h 40 min–7 h 10 min); standing, 5 h 10 min (range: 4 h 10 min–6 h 10 min); LPA, 2 h 10 min (range: 2 h–2 h 20 min); MVPA, 2 h 10 min (range: 1 h 40 min–2 h 20 min); sleeping, 8 h 20 min (range: 7 h 30 min–9 h).

Our investigation builds upon previous non-compositional analyses conducted with The Maastricht Study data by van der Berg et al [28, 29], extending these observational works by investigating optimal compositions and incorporating sleep time. The findings corroborate those of other observational analyses in populations with IGM and diabetes. Sedentary behaviour is adversely associated with cardiometabolic health [30, 31]. Less time spent being sedentary and more time spent participating in physical activity is associated with improved plasma glucose [32], insulin sensitivity [32–34], insulin levels, fat percentage, and triacylglycerol and cholesterol levels [34]. These studies largely suggest that MVPA is beneficial for cardiometabolic health, while acknowledging that reduction of sedentary time through the adoption of regular LPA is an important consideration irrespective of MVPA levels [35].

In alignment with the previous isotemporal analyses of data from The Maastricht Study [29], the findings suggest the viability of standing as a distinct alternative (along with physical activity and sleeping) to sitting, albeit with a greater amount required than for LPA and MVPA. These findings are in line with other compositional investigations that indicate that MVPA and stepping have the strongest associations with favourable cardiometabolic risk markers [7], including glucose and insulin [32]. Standing in CoDA has been less studied, with some evidence suggesting weak or mixed associations with health outcomes [36]. Optimal levels of standing time (4 h 10 min–6 h 10 min) have been demonstrated to be feasible in sedentary behaviour intervention settings [37]. Optimal sleep time (7 h 30 min–9 h) findings are aligned with current guidelines, which recommend a minimum of 7 h per day [38]. Interestingly, optimal sleeping levels differed slightly by health marker, especially for ISI-M, a marker of insulin sensitivity. Prolonged sleeping durations are associated with insulin resistance [39]; however, the optimal sleep duration must also be considered alongside beneficial associations of the ISI-M with standing and physical activity time. CoDA in this instance has balanced the benefit of sleeping with the benefit of longer time spent partaking in physical activity and longer standing time. Lastly, the inclusion of CMR provides clinical relevance to the findings. In the current study, the mean±SD difference between the average CMR estimate of the sample and the CMR estimate at the optimal composition was ~0.3±1.5. In a previous study, a similar CMR difference was found to be prospectively associated with significantly higher risk of cardiovascular events [40].

Experimental studies in people with type 2 diabetes that have acutely substituted sitting time with LPA have reported improvements in incremental AUC (iAUC) of glucose, triacylglycerol levels, insulin and insulin sensitivity [41]. Reducing sitting time through a combination of LPA and standing time has also been demonstrated to have positive effects on insulin sensitivity in postmenopausal women [42]. A review of field-based sedentary behaviour interventions determined that reductions in sitting corresponded with modest decreases in waist circumference, improvements to cardiometabolic risk (through systolic BP and HDL-cholesterol) and improved insulin sensitivity [43]. Across all reviewed trials, there were no changes in fasting glucose and HbA_1c_, possibly because there were limited studies featuring people with type 2 diabetes. These trials predominantly replaced sedentary time with standing, potentially leading to only modest associations observed with glycaemic outcomes [43]. Our findings were in line with those reported from experimental settings, where people with IGM benefited in terms of glucose and insulin from reductions in sedentary behaviour [44]. Further prospective evidence is required in free-living settings. Overall, current sedentary behaviour evidence suggests that, in addition to replacing sitting with standing time, sedentary behaviour interventions may need to incorporate more ambulatory behaviours to facilitate greater benefits in glucose metabolism, including those of higher intensity [45]. In line with this, it has been suggested that a ‘staircase’ approach could be considered when attempting to improve daily composition of waking behaviours, starting with replacing sedentary time with standing time, and then substituting in behaviours that are light intensity before more moderate-to-vigorous-intensity activities [46].

The findings from the present analysis could be used to further inform future iterations of time-use activity guidelines. Current 24 h activity guidelines [2] recommend specific quantities of time to be spent in MVPA (150 min/week), sedentary behaviour (<8 h/day) and sleep (7–9 h/day), but are less defined in their recommendations on how exactly sedentary behaviour should be replaced. The optimal zone upper limit of sedentary behaviour (7 h 10 min/day) supports these sedentary behaviour recommendations. Beneficial associations with cardiometabolic risk and glycaemic control were optimised as low as 4 h 10 min of standing per day and 2 h of LPA per day. These findings could help to inform future 24 h guidelines and provide evidence to inform recommendations pertaining to LPA and standing.

A key strength of this study is the use of CoDA in a large sample including those with type 2 diabetes, IGM and normoglycaemia for comparison purposes. Findings can be generalised to both sexes. All analyses were informed by data from a posture-sensing activity monitor that was able to accurately collect continuous measurements over multiple days. Notably, the current study is one of the few [29, 32, 36] to consider standing in a composition of time use. Few studies [32, 47] have ascertained the relationship between composition of daily behaviours with an array of risk markers indicative of subsequent disease risk, such as 2hPLG and ISI-M, which are resource-intensive to collect. The same is true for the sophisticated phenotyping that allows for appropriate adjustment of relevant confounders necessary in observational research. Observational studies have the potential to address novel hypotheses in the absence of more sophisticated prospective studies. However, limitations need to be considered. First, the sample includes individuals mainly of European descent and participants with well-controlled diabetes, therefore limiting generalisability of the findings to other populations. Second, the analyses are cross-sectional in nature, therefore precluding causal inference about the potential for composition changes to benefit risk markers. Similarly, the findings may be driven by reverse causation whereby poor glycaemic control, cardiometabolic ill-health or other comorbidities may be causing an increase in sedentary behaviours and a decrease in physical activity. Third, while the current analyses consider intensity of physical activity, this was based on stepping cadence cut points, warranting further investigation with more sophisticated measures of relative intensity such as heart rate. Finally, bout length (e.g. sedentary behaviours accumulated in prolonged bouts or activity accumulated in short bouts) was not considered, which might have distinct implications for cardiometabolic health that are potentially independent of total sitting time [48–50].

We provide novel observational evidence on compositional 24 h time use and an optimal balance of sitting time with standing, LPA, MVPA and sleeping. The optimal composition associated with all cardiometabolic risk and glycaemic control markers was as follows: sitting, 6 h (range: 5 h 40 min–7 h 10 min); standing, 5 h 10 min (range: 4 h 10 min–6 h 10 min); LPA, 2 h 10 min (range: 2 h–2 h 20 min); MVPA, 2 h 10 min (range: 1 h 40 min–2 h 20 min); and sleeping, 8 h 20 min (range: 7 h 30 min–9 h). These findings can help to inform future 24 h guidelines on sitting, standing, physical activity and sleep to improve cardiometabolic health and glycaemic control. For those with IGM or type 2 diabetes, our findings support recommendations to limit daily sedentary behaviour. However, longer-term and prospective study evidence, and intervention trials that change sedentary behaviour in daily time-use compositions, are needed to corroborate our findings.

### Supplementary Information

Below is the link to the electronic supplementary material.

Supplementary file1 (PDF 306 KB)

Supplementary file2 (HTM 1839 KB)

## Data Availability

Datasets generated and analysed are not publicly available but are available from the corresponding author upon reasonable request.

## Abbreviations

CMR: Clustered cardiometabolic risk score
CoDA: Compositional data analysis
FPG: Fasting plasma glucose
2hPLG: 2 h post-load glucose
IGM: Impaired glucose metabolism
ilr: Isometric log ratio
ISI-M: Matsuda index
LPA: Light-intensity physical activity
MVPA: Moderate-to-vigorous physical activity
NGM: Normoglycaemic metabolism
