# Supplementary material for: Associations of 24 h time-use compositions of sitting, standing, physical activity and sleeping with optimal cardiometabolic risk and glycaemic control: The Maastricht Study
Source: Diabetologia. 2024 Apr 24;67(7):1356–67. doi: 10.1007/s00125-024-06145-0 (PMC11153304; doi:10.1007/s00125-024-06145-0)
Supplement: Supplementary file 1 — Supplementary file1 (PDF 306 KB) [file 125_2024_6145_MOESM1_ESM.pdf]

ESM Table 1

Compositional isotemporal substitution for waist circumference

Substitution<br>+ 30 mins / -30 mins[a]	Overall<br>(n=2388)	NGM<br>(n=1341)	IGM<br>(n=363)	T2D<br>(n=684)
+Sit/ -Sleep Δ [95%CI]	0.01 [-0.01, 0.03]	0.01 [-0.01, 0.03]	0.03 [-0.02, 0.07]	0.01 [-0.02, 0.04]
+Sit/ -Stand Δ [95%CI]	**0.04 [0.03, 0.06]**	**0.04 [0.03, 0.06]**	0.01 [-0.03, 0.05]	**0.05 [0.02, 0.09]**
+Sit/ -LPA Δ [95%CI]	0 [-0.07, 0.07]	0 [-0.08, 0.08]	0.09 [-0.12, 0.3]	-0.03 [-0.2, 0.13]
+Sit/ -MVPA Δ [95%CI]	**0.29 [0.23, 0.36]**	**0.19 [0.12, 0.25]**	**0.3 [0.12, 0.47]**	**0.62 [0.42, 0.82]**
+Stand/ -Sit Δ [95%CI]	**-0.04 [-0.06, -0.03]**	**-0.04 [-0.06, -0.03]**	-0.01 [-0.05, 0.03]	**-0.05 [-0.08, -0.02]**
+Stand/ -Sleep Δ [95%CI]	**-0.03 [-0.05, -0.01]**	**-0.03 [-0.06, -0.01]**	0.02 [-0.04, 0.08]	**-0.04 [-0.08, -0.01]**
+Stand/ -LPA Δ [95%CI]	-0.04 [-0.12, 0.04]	-0.04 [-0.13, 0.05]	0.08 [-0.15, 0.31]	-0.08 [-0.26, 0.1]
+Stand/ -MVPA Δ [95%CI]	**0.25 [0.19, 0.31]**	**0.15 [0.08, 0.21]**	**0.29 [0.11, 0.47]**	**0.57 [0.37, 0.77]**
+LPA/ -Sit Δ [95%CI]	-0.01 [-0.05, 0.04]	-0.01 [-0.06, 0.05]	-0.06 [-0.19, 0.06]	0.01 [-0.08, 0.1]
+LPA/ -Sleep Δ [95%CI]	0 [-0.04, 0.05]	0 [-0.05, 0.06]	-0.04 [-0.17, 0.09]	0.02 [-0.08, 0.12]
+LPA/ -Stand Δ [95%CI]	0.04 [-0.01, 0.09]	0.04 [-0.02, 0.1]	-0.06 [-0.21, 0.1]	0.06 [-0.05, 0.18]
+LPA/ -MVPA Δ [95%CI]	**0.29 [0.2, 0.38]**	**0.18 [0.08, 0.28]**	0.23 [-0.03, 0.49]	**0.63 [0.38, 0.88]**
+MVPA/ -Sit Δ [95%CI]	**-0.15 [-0.19, -0.12]**	**-0.11 [-0.15, -0.07]**	**-0.16 [-0.25, -0.07]**	**-0.22 [-0.28, -0.15]**
+MVPA/ -Sleep Δ [95%CI]	**-0.14 [-0.18, -0.11]**	**-0.1 [-0.14, -0.06]**	**-0.14 [-0.24, -0.03]**	**-0.21 [-0.28, -0.14]**
+MVPA/ -Stand Δ [95%CI]	**-0.11 [-0.14, -0.08]**	**-0.07 [-0.11, -0.03]**	**-0.15 [-0.25, -0.06]**	**-0.16 [-0.23, -0.09]**
+MVPA/ -LPA Δ [95%CI]	**-0.16 [-0.24, -0.07]**	**-0.11 [-0.21, -0.01]**	-0.07 [-0.33, 0.19]	**-0.25 [-0.45, -0.04]**
+Sleep/ -Sit Δ [95%CI]	-0.01 [-0.03, 0.01]	-0.01 [-0.03, 0.01]	-0.03 [-0.07, 0.02]	-0.01 [-0.04, 0.02]
+Sleep/ -Stand Δ [95%CI]	**0.03 [0.01, 0.05]**	**0.04 [0.01, 0.06]**	-0.02 [-0.08, 0.05]	**0.05 [0.01, 0.09]**
+Sleep/ -LPA Δ [95%CI]	-0.01 [-0.09, 0.06]	-0.01 [-0.09, 0.08]	0.07 [-0.14, 0.28]	-0.04 [-0.21, 0.13]
+Sleep/ -MVPA Δ [95%CI]	**0.28 [0.22, 0.35]**	**0.18 [0.11, 0.25]**	**0.27 [0.09, 0.46]**	**0.61 [0.41, 0.81]**

[a] Denotes an increase in the first component, and a decrease in the second component.
Estimated difference in outcome with modification of composition by 30 minutes, estimates are reflective of predicted difference in z score from the sample mean z score.
Bold values indicates a statistically significant result whereby estimate confidence intervals do not overlap zero.
Models adjusted for age, sex, education, smoking status, and dietary intake score, the overall sample additionally adjusted for diabetes status.

ESM Table 2

Compositional isotemporal substitution for fasting plasma glucose

Substitution<br>+ 30 mins / -30 mins[a]	Overall<br>(n=2388)	NGM<br>(n=1341)	IGM<br>(n=363)	T2D<br>(n=684)
+Sit/ -Sleep Δ [95%CI]	0 [-0.01, 0.01]	0.01 [-0.01, 0.02]	0 [-0.02, 0.03]	0 [-0.04, 0.04]
+Sit/ -Stand Δ [95%CI]	0.01 [-0.01, 0.02]	0.01 [0, 0.02]	0 [-0.02, 0.03]	-0.01 [-0.04, 0.03]
+Sit/ -LPA Δ [95%CI]	**0.07 [0.01, 0.12]**	-0.01 [-0.05, 0.04]	-0.03 [-0.15, 0.08]	**0.3 [0.11, 0.49]**
+Sit/ -MVPA Δ [95%CI]	0 [-0.05, 0.05]	0 [-0.04, 0.03]	-0.05 [-0.15, 0.04]	-0.01 [-0.23, 0.22]
+Stand/ -Sit Δ [95%CI]	-0.01 [-0.02, 0]	**-0.01 [-0.02, 0]**	0 [-0.02, 0.02]	0.01 [-0.03, 0.04]
+Stand/ -Sleep Δ [95%CI]	-0.01 [-0.02, 0.01]	0 [-0.01, 0.01]	0 [-0.03, 0.03]	0.01 [-0.04, 0.05]
+Stand/ -LPA Δ [95%CI]	0.06 [0, 0.12]	-0.02 [-0.06, 0.03]	-0.03 [-0.16, 0.09]	**0.3 [0.09, 0.51]**
+Stand/ -MVPA Δ [95%CI]	-0.01 [-0.06, 0.04]	-0.01 [-0.05, 0.03]	-0.06 [-0.15, 0.04]	0 [-0.23, 0.23]
+LPA/ -Sit Δ [95%CI]	**-0.04 [-0.08, -0.01]**	0 [-0.02, 0.03]	0.02 [-0.05, 0.09]	**-0.17 [-0.28, -0.06]**
+LPA/ -Sleep Δ [95%CI]	**-0.04 [-0.08, 0]**	0.01 [-0.02, 0.04]	0.02 [-0.05, 0.09]	**-0.17 [-0.28, -0.06]**
+LPA/ -Stand Δ [95%CI]	-0.03 [-0.08, 0.01]	0.01 [-0.02, 0.04]	0.02 [-0.06, 0.11]	**-0.18 [-0.31, -0.05]**
+LPA/ -MVPA Δ [95%CI]	-0.04 [-0.12, 0.03]	0 [-0.05, 0.05]	-0.03 [-0.18, 0.11]	-0.17 [-0.46, 0.11]
+MVPA/ -Sit Δ [95%CI]	0 [-0.03, 0.02]	0 [-0.02, 0.02]	0.03 [-0.02, 0.08]	0 [-0.08, 0.07]
+MVPA/ -Sleep Δ [95%CI]	0 [-0.03, 0.03]	0 [-0.02, 0.03]	0.03 [-0.02, 0.08]	0 [-0.08, 0.07]
+MVPA/ -Stand Δ [95%CI]	0.01 [-0.02, 0.03]	0.01 [-0.01, 0.03]	0.03 [-0.02, 0.08]	-0.01 [-0.09, 0.07]
+MVPA/ -LPA Δ [95%CI]	0.07 [-0.01, 0.14]	-0.01 [-0.06, 0.05]	0 [-0.15, 0.14]	**0.29 [0.06, 0.53]**
+Sleep/ -Sit Δ [95%CI]	0 [-0.01, 0.01]	-0.01 [-0.02, 0.01]	0 [-0.03, 0.02]	0 [-0.04, 0.04]
+Sleep/ -Stand Δ [95%CI]	0.01 [-0.01, 0.02]	0 [-0.01, 0.02]	0 [-0.03, 0.03]	-0.01 [-0.05, 0.04]
+Sleep/ -LPA Δ [95%CI]	**0.07 [0.01, 0.13]**	-0.01 [-0.06, 0.03]	-0.03 [-0.15, 0.08]	**0.3 [0.1, 0.49]**
+Sleep/ -MVPA Δ [95%CI]	0 [-0.05, 0.05]	-0.01 [-0.04, 0.03]	-0.06 [-0.16, 0.04]	-0.01 [-0.23, 0.22]

[a] Denotes an increase in the first component, and a decrease in the second component.
Estimated difference in outcome with modification of composition by 30 minutes, estimates are reflective of predicted difference in z score from the sample mean z score.
Outcome underwent natural log transformation before being converted into z-score.
Bold values indicates a statistically significant result whereby estimate confidence intervals do not overlap zero.
Models adjusted for age, sex, education, smoking status, and dietary intake score, the overall sample additionally adjusted for diabetes status.

ESM Table 3

Compositional isotemporal substitution for 2h post load glucose

Substitution<br>+ 30 mins / -30 mins[a]	Overall<br>(n=2388)	NGM<br>(n=1341)	IGM<br>(n=363)	T2D<br>(n=684)
+Sit/ -Sleep Δ [95%CI]	0.01 [-0.01, 0.02]	0 [-0.01, 0.02]	-0.02 [-0.05, 0.01]	0.02 [-0.01, 0.05]
+Sit/ -Stand Δ [95%CI]	0 [-0.01, 0.01]	0.01 [0, 0.02]	0 [-0.03, 0.02]	-0.02 [-0.04, 0.01]
+Sit/ -LPA Δ [95%CI]	**0.1 [0.05, 0.16]**	**0.07 [0.02, 0.13]**	**0.17 [0.04, 0.3]**	**0.15 [0.02, 0.29]**
+Sit/ -MVPA Δ [95%CI]	0.01 [-0.04, 0.05]	0 [-0.04, 0.05]	-0.01 [-0.12, 0.1]	0.03 [-0.11, 0.17]
+Stand/ -Sit Δ [95%CI]	0 [-0.01, 0.01]	-0.01 [-0.02, 0]	0 [-0.02, 0.03]	0.01 [-0.01, 0.04]
+Stand/ -Sleep Δ [95%CI]	0 [-0.01, 0.02]	0 [-0.02, 0.01]	-0.01 [-0.05, 0.02]	0.03 [0, 0.06]
+Stand/ -LPA Δ [95%CI]	**0.1 [0.05, 0.16]**	**0.07 [0, 0.13]**	**0.18 [0.03, 0.32]**	**0.16 [0.02, 0.31]**
+Stand/ -MVPA Δ [95%CI]	0.01 [-0.04, 0.05]	0 [-0.05, 0.04]	-0.01 [-0.12, 0.11]	0.04 [-0.1, 0.19]
+LPA/ -Sit Δ [95%CI]	**-0.07 [-0.1, -0.04]**	**-0.05 [-0.09, -0.01]**	**-0.11 [-0.19, -0.03]**	**-0.09 [-0.17, -0.01]**
+LPA/ -Sleep Δ [95%CI]	**-0.06 [-0.09, -0.03]**	**-0.04 [-0.08, -0.01]**	**-0.12 [-0.21, -0.04]**	-0.07 [-0.16, 0.01]
+LPA/ -Stand Δ [95%CI]	**-0.07 [-0.1, -0.03]**	-0.04 [-0.08, 0]	**-0.11 [-0.2, -0.01]**	**-0.11 [-0.2, -0.01]**
+LPA/ -MVPA Δ [95%CI]	-0.06 [-0.12, 0]	-0.04 [-0.12, 0.03]	-0.11 [-0.28, 0.05]	-0.06 [-0.25, 0.13]
+MVPA/ -Sit Δ [95%CI]	-0.01 [-0.03, 0.02]	0 [-0.03, 0.02]	0 [-0.05, 0.06]	-0.02 [-0.07, 0.04]
+MVPA/ -Sleep Δ [95%CI]	0 [-0.02, 0.02]	0 [-0.03, 0.03]	-0.01 [-0.08, 0.05]	0 [-0.06, 0.06]
+MVPA/ -Stand Δ [95%CI]	-0.01 [-0.03, 0.02]	0 [-0.03, 0.03]	0 [-0.06, 0.06]	-0.03 [-0.09, 0.03]
+MVPA/ -LPA Δ [95%CI]	**0.1 [0.04, 0.16]**	0.07 [0, 0.14]	**0.18 [0.02, 0.34]**	0.13 [-0.03, 0.3]
+Sleep/ -Sit Δ [95%CI]	-0.01 [-0.02, 0.01]	0 [-0.02, 0.01]	0.02 [-0.01, 0.05]	-0.02 [-0.05, 0.01]
+Sleep/ -Stand Δ [95%CI]	0 [-0.02, 0.01]	0 [-0.01, 0.02]	0.01 [-0.03, 0.05]	-0.03 [-0.07, 0]
+Sleep/ -LPA Δ [95%CI]	**0.1 [0.05, 0.15]**	**0.07 [0.01, 0.13]**	**0.19 [0.06, 0.32]**	0.13 [0, 0.27]
+Sleep/ -MVPA Δ [95%CI]	0 [-0.04, 0.04]	0 [-0.05, 0.05]	0.01 [-0.11, 0.12]	0.01 [-0.13, 0.16]

[a] Denotes an increase in the first component, and a decrease in the second component.

Estimated difference in outcome with modification of composition by 30 minutes, estimates are reflective of predicted difference in z score from the sample mean z score.

Outcome underwent natural log transformation before being converted into z-score.

Bold values indicates a statistically significant result whereby estimate confidence intervals do not overlap zero.

Models adjusted for age, sex, education, smoking status, and dietary intake score, the overall sample additionally adjusted for diabetes status.

ESM Table 4

Compositional isotemporal substitution for HbA_{1c}

Substitution<br>+ 30 mins / -30 mins[a]	Overall<br>(n=2388)	NGM<br>(n=1341)	IGM<br>(n=363)	T2D<br>(n=684)
+Sit/ -Sleep Δ [95%CI]	0.01 [0, 0.03]	0 [-0.01, 0.02]	0.03 [0, 0.07]	0.03 [-0.01, 0.06]
+Sit/ -Stand Δ [95%CI]	-0.01 [-0.02, 0.01]	0 [-0.01, 0.01]	-0.01 [-0.03, 0.02]	**-0.03 [-0.07, 0]**
+Sit/ -LPA Δ [95%CI]	**0.07 [0.01, 0.13]**	-0.02 [-0.09, 0.04]	0.04 [-0.11, 0.18]	**0.31 [0.14, 0.49]**
+Sit/ -MVPA Δ [95%CI]	**0.06 [0.01, 0.11]**	0 [-0.05, 0.05]	-0.07 [-0.2, 0.05]	**0.24 [0.03, 0.45]**
+Stand/ -Sit Δ [95%CI]	0 [-0.01, 0.02]	0 [-0.01, 0.01]	0 [-0.02, 0.03]	0.03 [0, 0.06]
+Stand/ -Sleep Δ [95%CI]	**0.02 [0, 0.03]**	0 [-0.02, 0.02]	0.04 [-0.01, 0.08]	**0.06 [0.02, 0.1]**
+Stand/ -LPA Δ [95%CI]	**0.07 [0, 0.14]**	-0.02 [-0.09, 0.04]	0.04 [-0.12, 0.2]	**0.34 [0.15, 0.53]**
+Stand/ -MVPA Δ [95%CI]	**0.06 [0.01, 0.12]**	0 [-0.05, 0.05]	-0.07 [-0.19, 0.06]	**0.27 [0.06, 0.48]**
+LPA/ -Sit Δ [95%CI]	**-0.04 [-0.08, -0.01]**	0.02 [-0.02, 0.05]	-0.03 [-0.12, 0.06]	**-0.18 [-0.28, -0.08]**
+LPA/ -Sleep Δ [95%CI]	-0.03 [-0.07, 0.01]	0.02 [-0.02, 0.06]	0.01 [-0.09, 0.1]	**-0.15 [-0.26, -0.05]**
+LPA/ -Stand Δ [95%CI]	**-0.05 [-0.1, -0.01]**	0.02 [-0.03, 0.06]	-0.03 [-0.14, 0.08]	**-0.22 [-0.34, -0.1]**
+LPA/ -MVPA Δ [95%CI]	0.02 [-0.06, 0.09]	0.02 [-0.06, 0.09]	-0.1 [-0.28, 0.09]	0.06 [-0.21, 0.33]
+MVPA/ -Sit Δ [95%CI]	**-0.03 [-0.06, -0.01]**	0 [-0.03, 0.03]	0.03 [-0.03, 0.09]	**-0.09 [-0.16, -0.02]**
+MVPA/ -Sleep Δ [95%CI]	-0.02 [-0.05, 0.01]	0 [-0.03, 0.03]	0.06 [-0.01, 0.14]	-0.06 [-0.13, 0.01]
+MVPA/ -Stand Δ [95%CI]	**-0.04 [-0.07, -0.01]**	0 [-0.03, 0.03]	0.03 [-0.04, 0.09]	**-0.12 [-0.2, -0.05]**
+MVPA/ -LPA Δ [95%CI]	0.04 [-0.04, 0.11]	-0.03 [-0.1, 0.05]	0.07 [-0.12, 0.25]	**0.22 [0.01, 0.44]**
+Sleep/ -Sit Δ [95%CI]	-0.01 [-0.03, 0]	0 [-0.02, 0.01]	-0.03 [-0.07, 0]	-0.03 [-0.06, 0.01]
+Sleep/ -Stand Δ [95%CI]	**-0.02 [-0.04, 0]**	0 [-0.02, 0.02]	-0.04 [-0.08, 0.01]	**-0.06 [-0.1, -0.02]**
+Sleep/ -LPA Δ [95%CI]	0.06 [-0.01, 0.12]	-0.03 [-0.09, 0.04]	0 [-0.14, 0.15]	**0.28 [0.11, 0.46]**
+Sleep/ -MVPA Δ [95%CI]	0.05 [-0.01, 0.1]	0 [-0.05, 0.05]	-0.1 [-0.23, 0.03]	**0.21 [0, 0.42]**

[a] Denotes an increase in the first component, and a decrease in the second component.
Estimated difference in outcome with modification of composition by 30 minutes, estimates are reflective of predicted difference in z score from the sample mean z score.
Outcome underwent natural log transformation before being converted into z-score.
Bold values indicates a statistically significant result whereby estimate confidence intervals do not overlap zero.
Models adjusted for age, sex, education, smoking status, and dietary intake score, the overall sample additionally adjusted for diabetes status.

ESM Table 5

Compositional isotemporal substitution for Matsuda Index

Substitution<br>+ 30 mins / -30 mins[a]	Overall<br>(n=2388)	NGM<br>(n=1341)	IGM<br>(n=363)	T2D<br>(n=684)
+Sit/ -Sleep Δ [95%CI]	-0.02 [-0.04, 0]	-0.03 [-0.07, 0]	0 [-0.04, 0.05]	-0.01 [-0.04, 0.02]
+Sit/ -Stand Δ [95%CI]	0.02 [0, 0.04]	0.02 [-0.01, 0.05]	0 [-0.04, 0.04]	0.01 [-0.02, 0.04]
+Sit/ -LPA Δ [95%CI]	0.06 [-0.03, 0.15]	0.05 [-0.08, 0.18]	0.09 [-0.1, 0.27]	0.07 [-0.07, 0.21]
+Sit/ -MVPA Δ [95%CI]	**0.13 [0.05, 0.21]**	**0.17 [0.06, 0.27]**	0.11 [-0.05, 0.27]	0.09 [-0.06, 0.23]
+Stand/ -Sit Δ [95%CI]	-0.01 [-0.03, 0]	-0.02 [-0.05, 0.01]	0 [-0.04, 0.03]	-0.01 [-0.04, 0.02]
+Stand/ -Sleep Δ [95%CI]	**-0.03 [-0.06, -0.01]**	-0.05 [-0.09, -0.02]	0 [-0.06, 0.05]	-0.02 [-0.05, 0.02]
+Stand/ -LPA Δ [95%CI]	0.04 [-0.06, 0.14]	0.03 [-0.11, 0.18]	0.09 [-0.12, 0.29]	0.06 [-0.1, 0.22]
+Stand/ -MVPA Δ [95%CI]	**0.11 [0.04, 0.19]**	**0.15 [0.04, 0.26]**	0.11 [-0.05, 0.27]	0.08 [-0.07, 0.23]
+LPA/ -Sit Δ [95%CI]	-0.04 [-0.09, 0.02]	-0.03 [-0.11, 0.05]	-0.06 [-0.17, 0.06]	-0.04 [-0.12, 0.04]
+LPA/ -Sleep Δ [95%CI]	-0.06 [-0.11, 0]	-0.06 [-0.15, 0.02]	-0.06 [-0.17, 0.06]	-0.05 [-0.14, 0.04]
+LPA/ -Stand Δ [95%CI]	-0.02 [-0.09, 0.05]	-0.01 [-0.11, 0.09]	-0.05 [-0.19, 0.08]	-0.03 [-0.13, 0.07]
+LPA/ -MVPA Δ [95%CI]	0.09 [-0.02, 0.2]	0.14 [-0.02, 0.3]	0.06 [-0.18, 0.29]	0.04 [-0.15, 0.24]
+MVPA/ -Sit Δ [95%CI]	**-0.07 [-0.11, -0.03]**	**-0.09 [-0.15, -0.03]**	-0.06 [-0.14, 0.02]	-0.03 [-0.09, 0.02]
+MVPA/ -Sleep Δ [95%CI]	**-0.09 [-0.13, -0.04]**	**-0.12 [-0.19, -0.06]**	-0.06 [-0.15, 0.03]	-0.04 [-0.1, 0.02]
+MVPA/ -Stand Δ [95%CI]	**-0.05 [-0.09, -0.01]**	-0.07 [-0.13, 0]	-0.06 [-0.14, 0.03]	-0.03 [-0.09, 0.04]
+MVPA/ -LPA Δ [95%CI]	-0.01 [-0.12, 0.1]	-0.04 [-0.21, 0.12]	0.03 [-0.2, 0.26]	0.03 [-0.14, 0.21]
+Sleep/ -Sit Δ [95%CI]	0.02 [0, 0.04]	0.03 [0, 0.06]	0 [-0.05, 0.04]	0.01 [-0.02, 0.04]
+Sleep/ -Stand Δ [95%CI]	**0.03 [0.01, 0.06]**	**0.05 [0.01, 0.09]**	0 [-0.06, 0.06]	0.02 [-0.02, 0.05]
+Sleep/ -LPA Δ [95%CI]	0.08 [-0.02, 0.17]	0.08 [-0.05, 0.22]	0.09 [-0.1, 0.27]	0.08 [-0.07, 0.22]
+Sleep/ -MVPA Δ [95%CI]	**0.15 [0.07, 0.22]**	**0.2 [0.09, 0.31]**	0.11 [-0.05, 0.28]	0.09 [-0.06, 0.24]

[a] Denotes an increase in the first component, and a decrease in the second component.
Estimated difference in outcome with modification of composition by 30 minutes, estimates are reflective of predicted difference in z score from the sample mean z score.
Outcome underwent natural log transformation before being converted into z-score.
Bold values indicates a statistically significant result whereby estimate confidence intervals do not overlap zero.
Models adjusted for age, sex, education, smoking status, and dietary intake score, the overall sample additionally adjusted for diabetes status.

ESM Table 6

Compositional isotemporal substitution for clustered cardiometabolic risk.

Substitution<br>+ 30 mins / -30 mins[a]	Overall<br>(n=2388)	NGM<br>(n=1341)	IGM<br>(n=363)	T2D<br>(n=684)
+Sit/ -Sleep Δ [95%CI]	0.01 [0, 0.02]	0 [-0.01, 0.01]	0.01 [-0.02, 0.04]	0.02 [0, 0.04]
+Sit/ -Stand Δ [95%CI]	**0.02 [0.01, 0.03]**	**0.02 [0.01, 0.03]**	0 [-0.02, 0.03]	**0.02 [0, 0.04]**
+Sit/ -LPA Δ [95%CI]	**0.05 [0, 0.09]**	0.03 [-0.03, 0.08]	0.05 [-0.08, 0.17]	0.1 [0, 0.2]
+Sit/ -MVPA Δ [95%CI]	**0.12 [0.08, 0.15]**	**0.12 [0.07, 0.17]**	0.14 [0.03, 0.24]	**0.14 [0.02, 0.26]**
+Stand/ -Sit Δ [95%CI]	**-0.02 [-0.03, -0.01]**	**-0.02 [-0.03, -0.01]**	0 [-0.03, 0.02]	**-0.02 [-0.04, -0.01]**
+Stand/ -Sleep Δ [95%CI]	-0.01 [-0.02, 0]	**-0.02 [-0.04, 0]**	0.01 [-0.03, 0.04]	0 [-0.03, 0.02]
+Stand/ -LPA Δ [95%CI]	0.03 [-0.02, 0.08]	0.01 [-0.05, 0.07]	0.04 [-0.09, 0.18]	0.07 [-0.03, 0.18]
+Stand/ -MVPA Δ [95%CI]	**0.1 [0.06, 0.14]**	**0.1 [0.05, 0.15]**	**0.13 [0.03, 0.24]**	0.12 [0, 0.24]
+LPA/ -Sit Δ [95%CI]	**-0.03 [-0.06, 0]**	-0.02 [-0.06, 0.01]	-0.03 [-0.11, 0.04]	**-0.06 [-0.12, -0.01]**
+LPA/ -Sleep Δ [95%CI]	-0.02 [-0.05, 0.01]	-0.02 [-0.06, 0.02]	-0.02 [-0.1, 0.06]	-0.04 [-0.1, 0.02]
+LPA/ -Stand Δ [95%CI]	-0.01 [-0.04, 0.02]	0 [-0.04, 0.04]	-0.03 [-0.12, 0.06]	-0.04 [-0.1, 0.03]
+LPA/ -MVPA Δ [95%CI]	**0.08 [0.03, 0.14]**	**0.1 [0.03, 0.17]**	0.11 [-0.05, 0.26]	0.08 [-0.07, 0.23]
+MVPA/ -Sit Δ [95%CI]	**-0.06 [-0.08, -0.04]**	**-0.07 [-0.1, -0.04]**	**-0.07 [-0.13, -0.02]**	-0.06 [-0.1, -0.02]
+MVPA/ -Sleep Δ [95%CI]	**-0.05 [-0.07, -0.03]**	**-0.07 [-0.1, -0.04]**	**-0.06 [-0.12, 0]**	-0.04 [-0.08, 0]
+MVPA/ -Stand Δ [95%CI]	**-0.04 [-0.06, -0.02]**	**-0.05 [-0.08, -0.02]**	**-0.07 [-0.13, -0.01]**	-0.03 [-0.08, 0.01]
+MVPA/ -LPA Δ [95%CI]	**-0.02 [-0.07, 0.04]**	-0.04 [-0.11, 0.03]	-0.03 [-0.18, 0.13]	0.04 [-0.08, 0.16]
+Sleep/ -Sit Δ [95%CI]	-0.01 [-0.02, 0]	0 [-0.02, 0.01]	-0.01 [-0.04, 0.02]	**-0.02 [-0.04, 0]**
+Sleep/ -Stand Δ [95%CI]	0.01 [0, 0.02]	**0.02 [0.01, 0.04]**	-0.01 [-0.04, 0.03]	0.01 [-0.02, 0.03]
+Sleep/ -LPA Δ [95%CI]	0.04 [-0.01, 0.08]	0.03 [-0.03, 0.08]	0.04 [-0.09, 0.16]	0.08 [-0.02, 0.18]
+Sleep/ -MVPA Δ [95%CI]	**0.11 [0.07, 0.15]**	**0.12 [0.07, 0.17]**	**0.13 [0.02, 0.24]**	**0.12 [0, 0.24]**

[a] Denotes an increase in the first component, and a decrease in the second component.

Estimated difference in outcome with modification of composition by 30 minutes, estimates are reflective of predicted difference in CMR score from the sample mean score.

CMR score is a composite score including waist circumference, fasting plasma glucose, triaglycerol, HDL-cholesterol, and average blood pressure (systolic and diastolic).

Bold values indicates a statistically significant result whereby estimate confidence intervals do not overlap zero.

Models adjusted for age, sex, education, smoking status, and dietary intake score, the overall sample additionally adjusted for diabetes status.

ESM Table 7

Associations of time-use composition with glycaemic control and cardiometabolic risk markers

Model 1				Sitting[a]		Standing[a]		LPA[a]		MVPA[a]		Sleeping[a]	
	n	Adjusted R^2	Model p-value	β [95% CI][c]	p-value	β [95% CI][c]	p-value	β [95% CI][c]	p-value	β [95% CI][c]	p-value	β [95% CI][c]	p-value
WC	2388	0.44	<0.001	0.38 [0.23 - 0.53]	<0.001	-0.24 [-0.36 - -0.12]	<0.001	0.03 [-0.09 - 0.16]	0.598	-0.31 [-0.38 - -0.24]	<0.001	0.14 [-0.05 - 0.33]	0.153
FPG[c]	2387	0.63	<0.001	0.07 [-0.05 - 0.19]	0.266	-0.03 [-0.13 - 0.07]	0.582	-0.11 [-0.21 - -0.01]	0.035	0.01 [-0.05 - 0.06]	0.805	0.06 [-0.1 - 0.22]	0.448
2hPLG	2216	0.73	<0.001	0.12 [0.02 - 0.23]	0.026	0.05 [-0.04 - 0.14]	0.307	-0.18 [-0.27 - -0.09]	<0.001	0 [-0.05 - 0.05]	0.967	0.01 [-0.14 - 0.15]	0.935
HbA1c	2383	0.57	<0.001	0.15 [0.02 - 0.28]	0.027	0.12 [0.01 - 0.23]	0.028	-0.11 [-0.22 - 0]	0.056	-0.06 [-0.12 - 0]	0.056	-0.11 [-0.28 - 0.06]	0.224
ISI-M	2061	0.2	<0.001	0.03 [-0.17 - 0.22]	0.803	-0.13 [-0.3 - 0.03]	0.102	-0.1 [-0.26 - 0.06]	0.214	-0.15 [-0.25 - -0.06]	0.001	0.37 [0.11 - 0.62]	0.005
CMR	2384	0.55	<0.001	0.23 [0.13 - 0.32]	<0.001	-0.09 [-0.17 - -0.01]	0.026	-0.06 [-0.14 - 0.02]	0.123	-0.12 [-0.16 - -0.08]	<0.001	0.04 [-0.08 - 0.16]	0.515

WC = waist circumference; FPG = Fasting plasma glucose; 2hPLG = 2h glucose measure in oral glucose tolerance test; HbA1c = glycated haemoglobin; ISI-M = Matsuda Index described in methods; CMR = clustered cardiometabolic risk score.

[a] Coefficient for ilr1 parameter, representative of higher levels of selected behaviour relative to time in remaining behaviours.

[c] Outcomes transformed with natural logarithm.

Model 1 = adjusted for age, sex, education, smoking status, dietary intake score and diabetes status.

ESM Table 8

Modification by female sex on relationship of time-use composition with glycaemic control and cardiometabolic risk markers

	Sitting		Standing		LPA		MVPA		Sleeping	
	interaction [95% CI][c]	p-value	interaction [95% CI][c]	p-value	interaction [95% CI][c]	p-value	interaction [95% CI][c]	p-value	interaction [95% CI][c]	p-value
WC	0.09 [-0.21 - 0.39]	0.543	-0.12 [-0.37 - 0.13]	0.341	0.11 [-0.15 - 0.36]	0.406	-0.1 [-0.24 - 0.04]	0.406	0.02 [-0.37 - 0.41]	0.927
FPG	0.06 [-0.18 - 0.3]	0.622	-0.11 [-0.32 - 0.09]	0.264	0 [-0.21 - 0.21]	0.995	0.08 [-0.03 - 0.2]	0.995	-0.03 [-0.34 - 0.29]	0.853
2hPLG	-0.02 [-0.24 - 0.2]	0.846	-0.07 [-0.25 - 0.11]	0.452	0.05 [-0.13 - 0.24]	0.571	0.08 [-0.02 - 0.19]	0.571	-0.05 [-0.33 - 0.24]	0.755
HbA1c	-0.12 [-0.39 - 0.14]	0.357	-0.06 [-0.28 - 0.16]	0.602	0.03 [-0.2 - 0.25]	0.813	0.09 [-0.03 - 0.21]	0.813	0.07 [-0.28 - 0.41]	0.707
ISI-M	0.14 [-0.25 - 0.53]	0.492	0.17 [-0.15 - 0.5]	0.29	0 [-0.33 - 0.33]	0.981	-0.12 [-0.3 - 0.07]	0.981	-0.2 [-0.71 - 0.31]	0.443
CMR	0.04 [-0.14 - 0.23]	0.647	-0.18 [-0.34 - -0.03]	0.021	0.13 [-0.03 - 0.29]	0.121	-0.06 [-0.14 - 0.03]	0.121	0.07 [-0.18 - 0.31]	0.58

FPG = Fasting plasma glucose; 2hPLG = 2h glucose measure in oral glucose tolerance test; HbA1c = glycated haemoglobin; ISI-M = Matsuda Index described in methods; CMR = clustered cardiometabolic risk score.

Interaction by sex, coefficients are representative of females compared to males. Interaction term added to model to test moderation by females on the coefficient estimates (model 1). Model 1 = adjusted for age, education, smoking status, dietary intake score and diabetes status. Interaction terms included for ilr1, ilr2, ilr3 and ilr4.

ESM Table 9

Modification by IGM status on relationship of time-use composition with glycaemic control and cardiometabolic risk markers

	Sitting		Standing		LPA		MVPA		Sleeping	
	interaction [95% CI][c]	p-value	interaction [95% CI][c]	p-value	interaction [95% CI][c]	p-value	interaction [95% CI][c]	p-value	interaction [95% CI][c]	p-value
WC	0.06 [-0.36 - 0.48]	0.781	0.42 [0.05 - 0.79]	0.028	-0.26 [-0.64 - 0.11]	0.166	-0.16 [-0.37 - 0.06]	0.15	-0.06 [-0.37 - 0.06]	0.15
FPG	-0.07 [-0.41 - 0.27]	0.689	0.02 [-0.29 - 0.32]	0.914	0.01 [-0.29 - 0.32]	0.923	0.05 [-0.12 - 0.23]	0.548	-0.02 [-0.12 - 0.23]	0.548
2hPLG	-0.21 [-0.5 - 0.08]	0.156	0.18 [-0.08 - 0.44]	0.182	-0.25 [-0.51 - 0.01]	0.055	0.03 [-0.12 - 0.18]	0.698	0.26 [-0.12 - 0.18]	0.698
HbA1c	0.23 [-0.13 - 0.6]	0.211	0.14 [-0.18 - 0.47]	0.39	-0.11 [-0.43 - 0.22]	0.521	0.08 [-0.11 - 0.27]	0.404	-0.35 [-0.11 - 0.27]	0.404
ISI-M	0.09 [-0.44 - 0.61]	0.743	0.35 [-0.13 - 0.83]	0.151	-0.15 [-0.62 - 0.31]	0.521	0.12 [-0.15 - 0.38]	0.385	-0.41 [-0.15 - 0.38]	0.385
CMR	-0.03 [-0.29 - 0.24]	0.837	0.26 [0.02 - 0.5]	0.032	-0.11 [-0.35 - 0.12]	0.354	0.01 [-0.13 - 0.14]	0.894	-0.13 [-0.13 - 0.14]	0.894

FPG = Fasting plasma glucose; 2hPLG = 2h glucose measure in oral glucose tolerance test; HbA1c = glycated haemoglobin; ISI-M = Matsuda Index described in methods; CMR = clustered cardiometabolic risk score.

Interaction by diabetes status, IGM group compared to normoglycaemic group. Interaction term added to model to test moderation by IGM on the coefficient estimates (model 1). Model 1 = adjusted for age, sex, education, smoking status, dietary intake score. Interaction terms included for ilr1, ilr2, ilr3 and ilr4.

ESM Table 10

Supplementary Table. Modification by type 2 diabetes status on relationship of time-use composition with glycaemic control and cardiometabolic risk markers

	Sitting		Standing		LPA		MVPA		Sleeping	
	interaction [95% CI][c]	p-value	interaction [95% CI][c]	p-value	interaction [95% CI][c]	p-value	interaction [95% CI][c]	p-value	interaction [95% CI][c]	p-value
WC	-0.02 [-0.35 - 0.31]	0.914	0.09 [-0.17 - 0.35]	0.505	-0.03 [-0.3 - 0.24]	0.828	-0.14 [-0.29 - 0]	0.052	0.1 [-0.29 - 0]	0.052
FPG	0.1 [-0.17 - 0.37]	0.459	0.12 [-0.1 - 0.33]	0.284	-0.4 [-0.62 - -0.18]	<0.001	0.02 [-0.09 - 0.14]	0.705	0.16 [-0.09 - 0.14]	0.705
2hPLG	0.09 [-0.16 - 0.35]	0.473	0.18 [-0.03 - 0.38]	0.09	-0.06 [-0.27 - 0.15]	0.573	-0.02 [-0.13 - 0.09]	0.69	-0.19 [-0.13 - 0.09]	0.69
HbA1c	0.38 [0.09 - 0.67]	0.009	0.35 [0.11 - 0.58]	0.003	-0.43 [-0.67 - -0.19]	<0.001	-0.13 [-0.25 - 0]	0.051	-0.18 [-0.25 - 0]	0.051
ISI-M	-0.09 [-0.55 - 0.38]	0.716	0.27 [-0.09 - 0.63]	0.142	-0.07 [-0.44 - 0.3]	0.703	0.18 [-0.01 - 0.38]	0.068	-0.3 [-0.01 - 0.38]	0.068
CMR	0.09 [-0.11 - 0.3]	0.375	0.11 [-0.05 - 0.28]	0.184	-0.12 [-0.29 - 0.05]	0.175	0.1 [0 - 0.19]	0.041	-0.19 [0 - 0.19]	0.041

FPG = Fasting plasma glucose; 2hPLG = 2h glucose measure in oral glucose tolerance test; HbA1c = glycated haemoglobin; ISI-M = Matsuda Index described in methods; CMR = clustered cardiometabolic risk score.

Interaction by diabetes status, type 2 diabetes group compared to normoglycaemic group. Interaction term added to model to test moderation by IGM on the coefficient estimates. Model 1 = adjusted for age, sex, education, smoking status, dietary intake score. Interaction terms included for ilr1, ilr2, ilr3 and ilr4.

ESM Table 11

Associations of time-use composition with glycaemic control and cardiometabolic risk markers adjusting for waist circumference

Model 2				Sitting		Standing		LPA		MVPA		Sleeping	
	n	Adjusted R^2	Model p-value	β [95% CI][c]	p-value	β [95% CI][c]	p-value	β [95% CI][c]	p-value	β [95% CI][c]	p-value	β [95% CI][c]	p-value
FPG[c]	2387	0.64	<0.001	0.01 [-0.11 - 0.13]	0.85	0.01 [-0.09 - 0.11]	0.87	-0.11 [-0.21 - -0.01]	0.025	0.05 [0 - 0.11]	0.055	0.04 [-0.11 - 0.19]	0.618
2hPLG	2216	0.73	<0.001	0.1 [-0.01 - 0.21]	0.085	0.06 [-0.03 - 0.15]	0.17	-0.18 [-0.27 - -0.09]	<0.001	0.02 [-0.03 - 0.07]	0.432	0 [-0.14 - 0.14]	0.997
HbA1c	2383	0.58	<0.001	0.1 [-0.04 - 0.23]	0.154	0.16 [0.05 - 0.27]	0.004	-0.11 [-0.22 - 0]	0.043	-0.01 [-0.07 - 0.05]	0.661	-0.13 [-0.3 - 0.04]	0.135
ISI-M	2061	0.33	<0.001	-0.13 [-0.31 - 0.05]	0.153	-0.03 [-0.17 - 0.12]	0.738	-0.12 [-0.27 - 0.03]	0.105	-0.01 [-0.1 - 0.08]	0.814	0.29 [0.06 - 0.52]	0.015

FPG = Fasting plasma glucose; 2hPLG = 2h glucose measure in oral glucose tolerance test; HbA1c = glycated haemoglobin; ISI-M = Matsuda Index described in methods.
[a] Coefficient for ilr1 parameter, representative of higher levels of selected behaviour relative to time in remaining behaviours.
[c]Outcomes transformed with natural logarithm.
Model 2 = Model 1 + adjusted for waist circumference, except the waist circumference outcome itself and CMR which were comprised of waist circumference.

ESM Table 12

Compositions of 24 h time use associated with the most optimum levels of glycaemic control and cardiometabolic risk markers in normoglycaemic group

Measure (z- score)	Compositional centre of behaviour				
Sample mean, h:min (1st – 99th percentile range) [a]	Sitting 9:00 (5:40 - 12:40)	Standing 4:20 (2:10 - 7:40)	LPA 1:00 (0:30 - 2:20)	MVPA 0:50 (0:20 – 2:30)	Sleeping 8:10 [6:20 - 10:30)
Waist circumference	6:20 (5:40 - 8:40)	6:40 (5:00 - 7:40)	1:10 (0:30 - 2:20)	2:10 (1:30 - 2:30)	7:20 (6:20 - 10:20)
FPG	6:20 (5:40 - 8:30)	6:40 (4:40 - 7:40)	0:50 (0:30 - 2:20)	0:50 (0:20 - 2:30)	9:00 (6:40 - 10:30)
2h PLG	6:20 (5:40 - 8:10)	6:0 (2:50 - 7:40)	2:10 (1:50 - 2:20)	1:10 (0:20 - 2:30)	8:00 (6:20 - 10:30)
HbA_{1c}	8:00 (5:40 - 12:10)	4:30 (2:10 - 7:40)	0:30 (0:30 - 0:40)	1:10 (0:20 - 2:30)	9:20 (7:40 - 10:30)
ISI-M	7:10 (5:40 - 10:10)	6:10 (3:00 - 7:40)	1:40 (0:30 - 2:20)	2:10 (1:10 - 2:30)	6:40 (6:20 - 7:50)
CMR	6:20 (5:40 - 8:20)	6:30 (4:20 - 7:40)	1:30 (0:30 - 2:20)	2:10 (1:20 - 2:30)	7:10 (6:20 - 9:20)
Overlapped optimal zone [b]	5:40 (5:40 - 5:50)	7:30 (7:00 - 7:40)	1:40 (1:40 - 2:00)	1:40 (1:20 - 2:00)	7:20 (7:00 - 7:50)

Data correspond to the compositional centre and range of the most-optimal (top 5%) compositions. Compositional centre was calculated within the most-optimal compositional area using the geometric mean of the five behaviour components (sitting, standing, LPA, MVPA and sleeping) for the NGM sample.

Data are presented as h:min (range), unless stated otherwise, with estimates rounded to the nearest 10 min. This may have resulted in combined estimates not forming exactly 24 h

All models are adjusted for age, sex, education, smoking status, dietary intake score and diabetes status

[a]Shows the geometric mean of the sample in h:min, as well as the range of compositions in the study footprint including time use from the first to the 99th percentile for each behaviour

[b]Compositional centre of the overlapped optimal zone required extending all compositions (from the optimum top 5%) to the optimum top 10%, to obtain data on mutual overlap between markers

ESM Table 13

Compositions of 24 h time use associated with the most optimum levels of glycaemic control and cardiometabolic risk markers in impaired glucose metabolism group

Measure (z- score)	Compositional centre of behaviour				
Sample mean, h:min (1st – 99th percentile range) [a]	Sitting 9:20 (5:30 - 13:00)	Standing 4:10 (2:10 - 8:20)	LPA 1:00 (0:30 - 2:20)	MVPA 0:50 (0:10 – 2:10)	Sleeping 8:10 (6:30 - 10:20)
Waist circumference	6:20 (5:30 - 9:20)	4:30 (2:10 - 8:20)	1:50 (0:40 - 2:20)	2:00 (1:20 - 2:10)	9:00 (6:30 - 10:20)
FPG	8:40 (5:30 - 13:00)	5:10 (2:10 - 8:20)	0:50 (0:30 - 1:30)	0:10 (0:10 - 0:20)	8:30 (6:30 - 10:20)
2h PLG	9:00 (5:30 - 13:00)	4:20 (2:10 - 8:20)	2:10 (1:50 - 2:20)	0:50 (0:10 - 2:10)	7:00 (6:30 - 8:30)
HbA_{1c}	7:50 (5:30 - 13:00)	4:00 (2:10 - 8:20)	1:40 (0:30 - 2:20)	0:10 (0:10 - 1:50)	9:40 (7:50 - 10:20)
ISI-M	6:50 (5:30 - 11:10)	4:40 (2:10 - 8:20)	2:10 (1:30 - 2:20)	2:00 (1:20 - 2:10)	8:00 (6:30 - 10:20)
CMR	6:30 (5:30 - 10:00)	4:30 (2:10 - 8:20)	2:00 (0:50 - 2:20)	2:00 (1:20 - 2:10)	8:40 (6:30 - 10:20)
Overlapped optimal zone[#]	7:00 (5:30 - 10:20)	5:00 (2:10 - 8:20)	2:20 (2:00 - 2:20)	2:00 (1:20 - 2:10)	7:10 (6:30 - 8:40)

Data correspond to the compositional centre and range of the most-optimal (top 5%) compositions. Compositional centre was calculated within the most-optimal compositional area using the geometric mean of the five behaviour components (sitting, standing, LPA, MVPA and sleeping) for the IGM sample.

Data are presented as h:min (range), unless stated otherwise, with estimates rounded to the nearest 10 min. This may have resulted in combined estimates not forming exactly 24 h

All models are adjusted for age, sex, education, smoking status, dietary intake score and diabetes status

[a]Shows the geometric mean of the sample in h:min, as well as the range of compositions in the study footprint including time use from the first to the 99th percentile for each behaviour

[b]Compositional centre of the overlapped optimal zone required extending all compositions (from the optimum top 5%) to the optimum top 10%, to obtain data on mutual overlap between markers

ESM Table 14

Compositions of 24 h time use associated with the most optimum levels of glycaemic control and cardiometabolic risk markers in type 2 diabetes group

Measure (z- score)	Compositional centre of behaviour				
Sample mean, h:min ($1^{st} – 99^{th}$ percentile range) [a]	Sitting 10:00 (6:10 - 14:00)	Standing 3:40 (1:20 - 7:50)	LPA 0:50 (0:20 - 2:10)	MVPA 0:30 (0:10 – 2:00)	Sleeping 8:20 (6:10 - 11:00)
Waist circumference	7:00 (6:10 - 10:10)	6:40 (4:10 - 7:50)	0:50 (0:20 - 2:10)	1:50 (1:00 - 2:00)	7:30 (6:10 - 11:00)
FPG	9:40 (6:10 - 14:00)	2:10 (1:20 - 7:50)	2:10 (1:50 - 2:10)	1:00 (0:10 - 2:00)	8:30 (6:10 - 11:00)
2h PLG	8:50 (6:10 - 12:50)	1:50 (1:20 - 4:10)	1:50 (1:00 - 2:10)	1:10 (0:10 - 2:00)	9:50 (6:10 - 11:00)
HbA_{1c}	9:00 (6:10 - 13:20)	1:50 (1:20 - 4:20)	1:50 (1:00 - 2:10)	1:30 (0:10 - 2:00)	9:30 (6:10 - 11:00)
ISI-M	7:40 (6:10 - 11:30)	5:50 (2:10 - 7:50)	1:50 (1:00 - 2:10)	1:30 (0:30 - 2:00)	6:50 (6:10 - 9:00)
CMR	6:30 (6:10 - 7:50)	5:30 (2:00 - 7:50)	1:50 (1:00 - 2:10)	1:30 (0:30 - 2:00)	8:20 (6:10 - 11:00)
Overlapped optimal zone[#]	6:20 (6:10 - 6:40)	4:30 (4:10 - 5:00)	2:10 (2:10 - 2:10)	2:00 (1:40 - 2:00)	9:00 (8:40 - 9:30)

Data correspond to the compositional centre and range of the most-optimal (top 5%) compositions. Compositional centre was calculated within the most-optimal compositional area using the geometric mean of the five behaviour components (sitting, standing, LPA, MVPA and sleeping) for the type 2 diabetes sample.

Data are presented as h:min (range), unless stated otherwise, with estimates rounded to the nearest 10 min. This may have resulted in combined estimates not forming exactly 24 h

All models are adjusted for age, sex, education, smoking status, dietary intake score and diabetes status

[a]Shows the geometric mean of the sample in h:min, as well as the range of compositions in the study footprint including time use from the first to the 99th percentile for each behaviour

[b]Compositional centre of the overlapped optimal zone required extending all compositions (from the optimum top 5%) to the optimum top 10%, to obtain data on mutual overlap between markers

ESM Figure 1

Optimal compositions of time use visualized in multidimensional space.

ESM Fig. 1 supplied as separate HTML file.

Interactive four-axis quaternary tetrahedron plot depicting overlapping optimal compositions.

Sleeping time was fixed to 8 h. Each corner of the tetrahedron depicts 100% of time use, and a composition data point directly in the middle of the tetrahedron would depict equal, 25% (4 h) of time use in sitting, standing, LPA, and MVPA.

Pink area denotes compositions of time use associated with optimal (top 5%) fasting plasma glucose. Blue area denotes the same for waist circumference. Red area is the mutual overlapping area of fasting plasma glucose, waist circumference, as well as the remaining health outcomes: 2h PLG, HbA_{1c}, ISI-M, and CMR (not shown). Red area represents the overlapped optimal zone.

Figure can be rotated by clicking and moving the mouse. Zooming in and out can be performed with the second mouse button or with the mouse wheel.
