# Supplementary material for: Associations of 24 h time-use compositions of sitting, standing, physical activity and sleeping with optimal cardiometabolic risk and glycaemic control: The Maastricht Study
Source: Diabetologia. 2024 Apr 24;67(7):1356–67. doi: 10.1007/s00125-024-06145-0 (PMC11153304; doi:10.1007/s00125-024-06145-0)
Supplement: Supplementary file 2 — Supplementary file2 (HTM 1839 KB) [file 125_2024_6145_MOESM2_ESM.htm]

rglWebGL
  
  
  
  
  
  
  
  
  
  
  
  
  
  
  
  
